# Thermodiluted relative tidal volume estimation using a thermal camera in operating room under spinal anesthesia

**DOI:** 10.1186/s12938-022-01028-0

**Published:** 2022-09-07

**Authors:** JunHwan Kwon, Oyun Kwon, KyeongTeak Oh, Jeongmin Kim, Cheung Soo Shin, Sun K. Yoo

**Affiliations:** 1 Department of Medical Engineering, Yonsei University College of Medicine, Seoul, Republic of Korea; 2 Department of Anesthesiology and Pain Medicine, Severance Hospital, Anesthesia and Pain Research Institute, Yonsei University College of Medicine, Seoul, Republic of Korea

**Keywords:** Respiration, Tidal volume, Thermal camera, Non-contact

## Abstract

**Background:**

Estimating relative tidal volume is an important factor when monitoring breathing status. The relationship between temperature and respiration volume has rarely been studied. In this paper, a formula was derived for calculating thermodiluted respiration volume from temperature changes in the nasal cavity. To evaluate the proposed formula, the study compared the relative tidal volume estimated by the proposed formula with that recorded by a respiration volume monitor (Exspiron1Xi, RVM). Thermal data were obtained for 8 cases at a rate of 10 measurements per second. Simultaneous recordings by the RVM are regarded as the reference.

**Results:**

The mean of ICC coefficient is 0.948 ± 0.030, RMSE is 0.1026 ± 0.0284, R-squared value is 0.8962 ± 0.065 and linear regression coefficient $\mathrm{\alpha }$ is 0.966 ± 0.104, $\upbeta$ is 0.042 ± 0.057. Bland–Altman plot showed 96.01% of samples that the difference between the measured and estimated values exists within 2 standard deviations.

**Conclusions:**

In this paper, a model that can thermodynamically calculate the relationship between thermal energy and respiration volume is proposed. The thermodiluted model is a feasible method for estimating relative respiration tidal volumes.

## Background

Breathing is one of the characteristics that reflect the human physiological state. Breathing is the process of supplying oxygen to the body and is the process of moving oxygen-containing air from outside to inside the body. Measuring breathing consists of monitoring a person’s breathing pattern by measuring respiration volume, and respiration rate. Among these parameters, respiration volume is the air quantity that a person supplies to their body, and it is used as an important biosignal from which the oxygen supply can be inferred [1]. Therefore, it is important to measure respiration volume for the vital sign, and past researchers have suggested a variety of methods to measure this variable.

In general, respiration volume is determined by directly measuring the physical changes that occur when breathing or by indirect characteristics that are affected by these changes. Methods for directly measuring airflow include face masks [2] and spirometry [3]. In this method, the respiration volume is calculated by directly measuring the airflow of the person by using an airflow meter. The method for indirectly measuring the flow of air by measuring the partial pressure of carbon dioxide in the air is called capnography [4, 5]. Another method uses the TIG (thoracic impedance gradient) [6, 7] to measure the changes in impedance that occur with the movement of the air-contained rib cage due to breathing. However, the above methods are limited by the inconvenience of attaching sensors to the subject and may lead to infection in severe cases. In particular, the TIG method can continuously measure respiration volume, but it cannot be used when it interferes with body impedance during measurement as with an electrosurgical unit (ESU) and is sensitive to the movements of the subject. In this paper, the formula was proposed for estimating thermodiluted relative tidal volume using a thermal camera.

A thermal camera is used as a non-contact method and can eliminate the discomfort and risk of infection that may occur due to physical contact. A thermal camera can measure the energy radiated from an object (or living things) by using an infrared (IR) detector. Such IR detectors are known to be useful for measuring vital signs, including respiratory activity [8]. Moreover, research on respiration rates has shown that temperature changes in the nasal cavity and breathing affect each other [9–12]. Fei J and Pavlidis I (2007) [9] proposed a virtual thermistor that used a coalitional tracking algorithm and wavelet decomposition to extract maximum frequency. They estimate respiration rate by using temperature changes near a nostril. Jin F and Pavlidis I (2010) [10] proposed an investigative tool to understand physiology by estimating breathing information using the mean thermal signal of the nostril and wavelet analysis. Pereira CB et al. (2015) [11] extract breathing rate (BR) to estimate abnormal states by using the thermal camera in healthy people. Pereira CB et al. (2019) [12] proposed respiratory rate monitoring algorithm using “black-box” that does not rely on tracking specific anatomic landmarks in newborn infants.

The respiration volume measurement method, which uses a thermal camera, indicates respiration volume by measuring the small temperature changes that occur in the nasal cavity due to breathing. In this study, a thermal camera was used to measure temperature changes, and a proposed formula for the respiration volume from the temperature change was created by adding the concept of thermodilution to the breathing model [13]. In our study, the model for estimating relative tidal volume to be re-interpreted in a semi-analytic approach was proposed. The proposed model is constructed to be more analytical than previous studies by observing the thermo-dynamic properties of respiration and applying them to thermodilution. Using the proposed model, trends in relative respiration volume can be estimated in the stable surgical environment. Also, in the operating room, there are not only several contact infections, but also people who complain of contact discomfort caused by allergies and burns. Estimating relative respiration volume using thermal camera is free from infection, discomfort of contact method and inference of ESU. The proposed method for estimating respiration volume with a thermal camera is based on the temperature change inside the nasal cavity that occurs when a person breathes. In the thermal image obtained from the thermal camera, the raw signal is extracted by aligning and separating the temperature change region due to breathing and calculating the temperature difference for the region of interest (ROI). The noise-sensitive raw signal is stabilized using an ensemble continuous wavelet transform (CWT) to extract the respiratory period index. Based on the extracted index, the relative tidal volume is obtained by calculating a raw signal value using the proposed formula. In the model and formula section of this paper, the similarities, and differences between the proposed model and thermodilution for cardiac output are described, and the closed system is defined. The formula was derived for estimating the relative tidal volume using measures of thermal energy. The experimental conditions and methods are presented**.** The relative tidal volume that is estimated using the derived formula and the TIG values used as a reference are compared in the results**.** The Discussion section describes the meaning of the proposed model and limitations**.** Finally, the Conclusion presents the results and meaning of this paper.

## Results

### Comparison of the correlation of tidal volumes as estimated by the thermodiluted volume and TIG

**Fig. 1:**
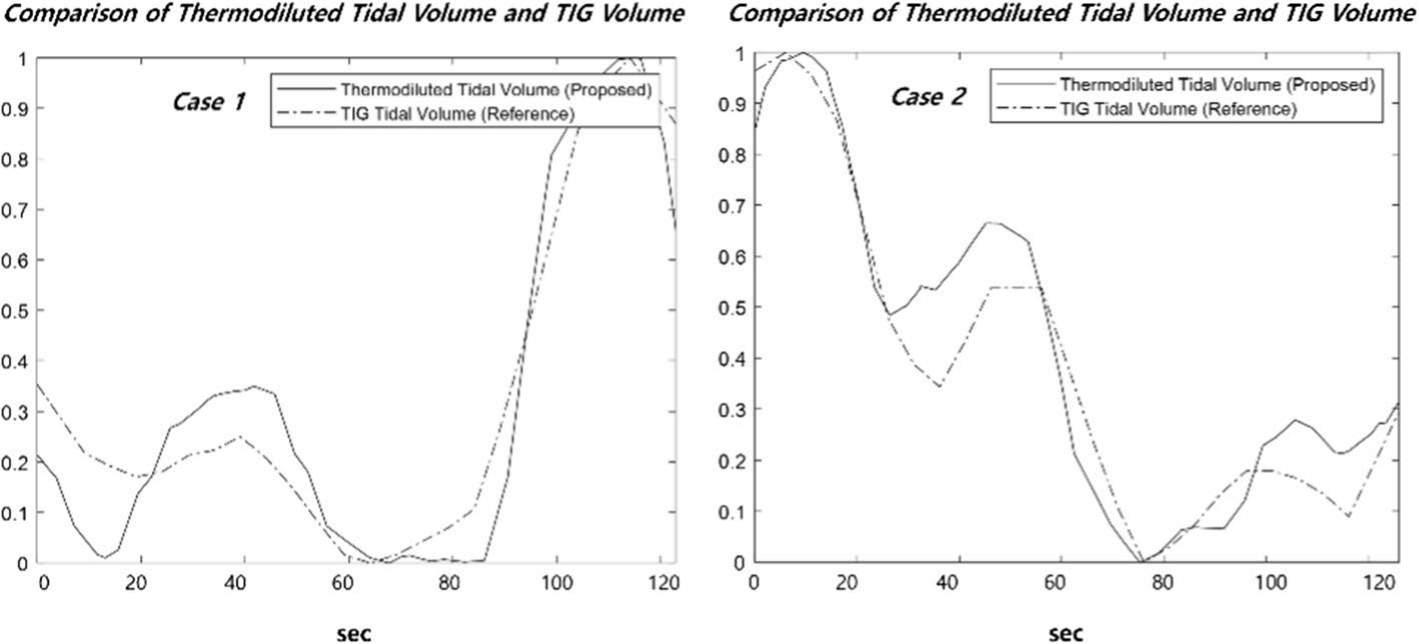
Comparison of thermodiluted tidal volume and TIG tidal volume

The proposed formula was used to compare the degree of linearity between relative tidal volumes that were calculated using data from the thermal imaging camera and relative tidal volumes that were calculated using data from the impedance device. The part with a change in respiration volume was selected, and a total of eight cases per minute were compared. Figure 1 shows a case in the graph that compares mean relative tidal volumes determined using TIG and those determined using the proposed formula that thermodiluted model from thermal imaging camera data. The results of the visual inspection show that the trends of the respiration volume graphs are similar. These results showed that the relative tidal volume obtained from the proposed model is well estimated from the actual patient state under spinal anesthesia.

### Statistical values of correlation between thermodiluted and TIG relative tidal volume

Table 1 represents the Intra-Class Correlation coefficient (ICC, C-1) value, root mean square error and linear regression coefficient value of each case. Table 1 shows that the proposed model has a high ICC correlation coefficient value. This means that the relative volume calculated from the proposed model, which is a non-contact method, has a high correlation coefficient with the contact-method reference. And linear regression R-squared value also has a high coefficient value. The mean of ICC coefficient is 0.9480 ± 0.030, RMSE is 0.1026 ± 0.0284, R-squared value is 0.8962 ± 0.065 and linear regression coefficient $\mathrm{\alpha }$ is 0.966 ± 0.104 $\upbeta$ is 0.042 ± 0.057. In particular, the values $\mathrm{\alpha }$ and $\upbeta$ of each case have similar values. The meaning of these values means that the proposed model is applied efficiently regardless of the case in a limited environment.

**Table 1:** Statistical values of correlation between thermodiluted and TIG relative to tidal volume

Case	ICC coefficient	RMSE	Linear regression R-squared value (coefficient)
Case 1	0.9556	0.0963	0.92 ($\mathrm{\alpha }$=0.89, $\upbeta$ =0.04)
Case 2	0.9626	0.0882	0.93 ($\mathrm{\alpha }$=0.93, $\upbeta$ =-0.01)
Case 3	0.9768	0.0767	0.96 ($\mathrm{\alpha }$=1.01, $\upbeta$ =-0.04)
Case 4	0.9853	0.0589	0.98 ($\mathrm{\alpha }$=1.07, $\upbeta$ =0.01)
Case 5	0.9393	0.1255	0.81 ($\mathrm{\alpha }$=1.06, $\upbeta$ =0.09)
Case 6	0.9421	0.1350	0.90 ($\mathrm{\alpha }$=1.07, $\upbeta$ =0.05)
Case 7	0.8871	0.1379	0.80 ($\mathrm{\alpha }$=0.78, $\upbeta$ =0.14)
Case 8	0.9349	0.1024	0.87 ($\mathrm{\alpha }$=0.92, $\upbeta$ =0.06)

**Fig. 2:**
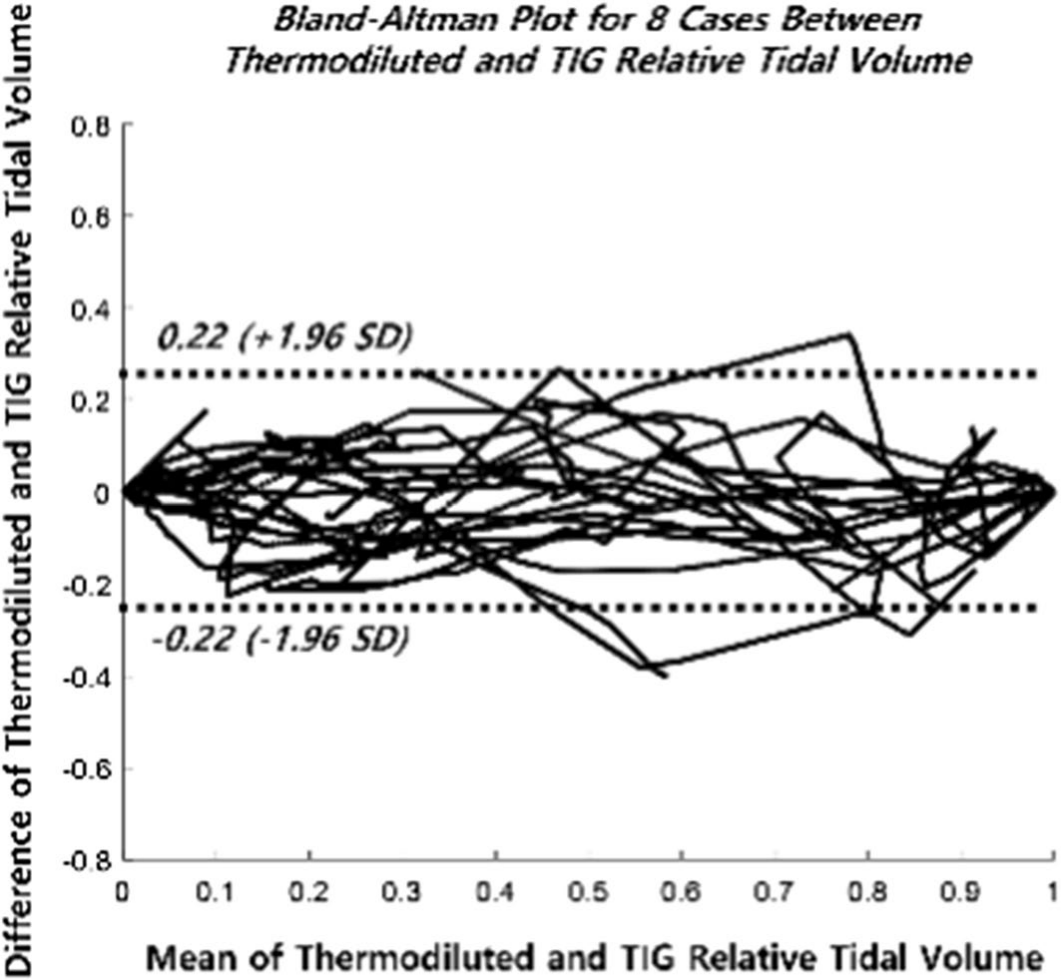
Bland–Altman plot between thermal camera and TIG relative tidal volume

**Fig. 3:**
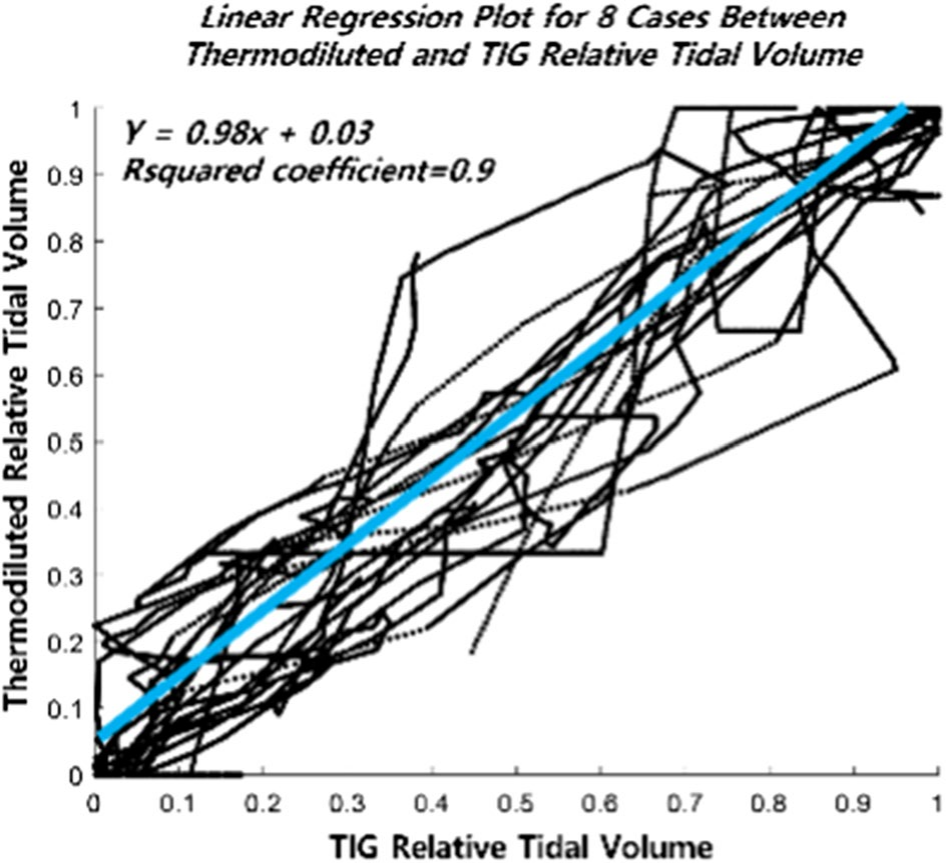
Linear regression plot between thermal camera and TIG relative tidal volume

Figure 2 shows the Bland–Altman plot for all cases between thermodiluted and TIG relative tidal volume. Bland–Altman plot showed 96.01% of samples that the difference between the measured and estimated values exists within 2 standard deviations. Figure 3 shows linear regression for all cases between thermodiluted and TIG relative tidal volume. R-squared value is 0.90, $\mathrm{\alpha }$ is 0.98, and $\upbeta$ is 0.03.

### Comparison of the thermodiluted volume and pulse oximetry saturation (Sp02)

**Fig. 4:**
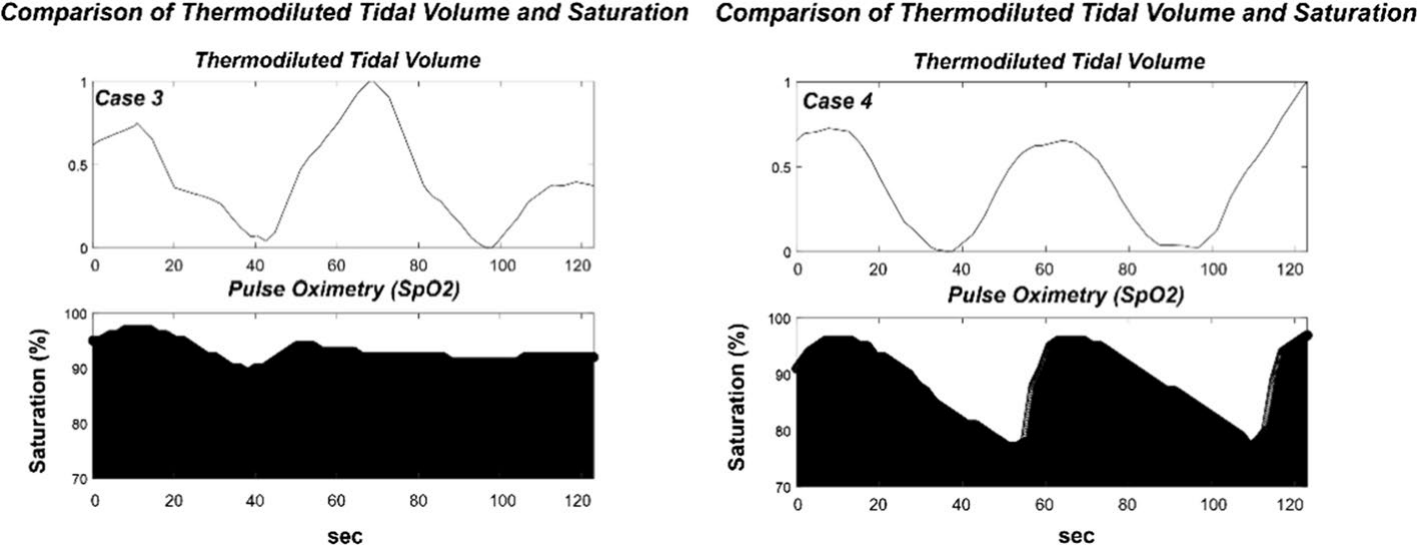
Comparison of the thermodiluted volume and pulse oximetry saturation

Figure 4 shows a case in the graph that compares mean relative tidal volumes determined using the proposed formula that thermodiluted model from the thermal camera and oxygen saturation from pulse oximetry. When the relative tidal volume using thermodiluted model decreased, the level of oxygen saturation decreased. Figure 4 shows that the proposed model reflects the blood oxygen saturation.

## Discussion

In this paper, a model that can thermodynamically calculate the relationship between thermal energy and respiration volume is proposed. The results show a strong linear correlation between relative tidal volumes estimated as thermal energy and those obtained as TIGs. The results of Fig. 2 compare the trends of estimated tidal volumes from the reference and proposed models. Because of the low resolution, the TIG values do not represent all changes, as they are interpolated values, but it can be seen that the trend is strongly correlated with the values estimated by the proposed formula. Table 1 shows the results of linear regression and ICC coefficient between the reference and proposed model, which show that the proposed model has a strong linear relationship with the values obtained by TIG. Figure 3, Bland–Altman plot, shows 96.01% of samples that the difference between the measured and estimated values exists within 2 standard deviations. The proposed model is a non-contact diagnostic tool for low respiration relative tidal volume in subjects who are susceptible to hypoxemia during monitored anesthesia care.

In other words, these results are estimated adequately by the proposed model. These results mean that if body temperature ${(T}_{L})$ and nose temperature ${(T}_{N})$ were given, the proposed formula can meaningfully estimate non-contact relative respiration volume.

This paper proposes a feasible method for estimating respiration volumes with no patient contact. The method of using contact can cause patient discomfort and infection. In particular, TIG is interfered with by electrical surgical devices. However, many experiments for the proposed model will increase usability in various environments, such as shape, distance, angle, FOV, camera specification, etc. In practice, body temperature ${(T}_{L})$ is not always the same for each breathing cycle, there is a fine difference. Body temperature ${(T}_{L})$ is highly affected by the depth of the respiration and how long the respiration-related air stays in the lung. Also, depending on the respiration pattern, ${Q}_{N}$ and ${Q}_{L}$ are not always constant. These limitations should be improved through experiments on various environments based on the proposed basic model.

Nevertheless, the proposed model is meaningful in that it physiologically describes temperature change of respiration-related air, and presents a basic model to estimate non-contact respiration relative volume under limited conditions.

This contactless respiratory measurement method will improve performing significant respiratory monitoring in operating rooms, intensive care units (ICUs), and isolation wards to treat infectious patients. It is also possible to measure respiration rates and respiration patterns. Respiration relative volumes were estimated using a thermal camera in a stable environment. When using a thermal camera, estimating respiration relative volumes has limitations that involve sensitivity to registration. If there are changes in the ROI area that obtains temperatures, the characteristics of surface errors can change significantly because the thermal camera receives data that are projected onto a 2-D plane. In this study, the ideal model was proposed, and errors from measurements were assumed. All cases were measured in a stable state with respiration changes during spinal anesthesia in the operation room. The limitation of this study is that the experiment did not consider various environment variables. The experiment was performed in the operation room environment where the environment temperature was stable. Based on the basic model, follow-up studies are needed to apply it to various environments and conditions.

## Conclusions

In this paper, a formula was proposed to determine respiration volumes by measuring thermal energy. By applying changes in thermal energy to the respiratory system, a linear relationship for relative tidal volume was derived. When comparing tidal volumes determined using the derived equation and using the RVM, the linear correlation R-squared value averaged 0.89, and the variance was 0.065. The mean of ICC coefficient is 0.948 ± 0.030, RMSE is 0.1026 ± 0.0284 and linear regression coefficient $\mathrm{\alpha }$ is 0.966 ± 0.104 $\upbeta$ is 0.042 ± 0.057. When estimating respiration relative volumes, it is advantageous to determine respiration volumes in a non-contact manner for better patient safety and comfort. In this paper, the study demonstrated that it is possible to improve on the existing contact method of measuring respiration volumes by deriving a formula based on differences in thermal energy recorded by a thermal imaging camera to determine respiratory volumes. To obtain accurate temperature differences, the area of temperature change must exist in the thermal camera’s field of view. In addition, the movement needs to be corrected for long-term monitoring. In future work, the proposed model should be improved to estimate respiration volumes in various environments.

## Methods

### The model

**Fig. 5:**
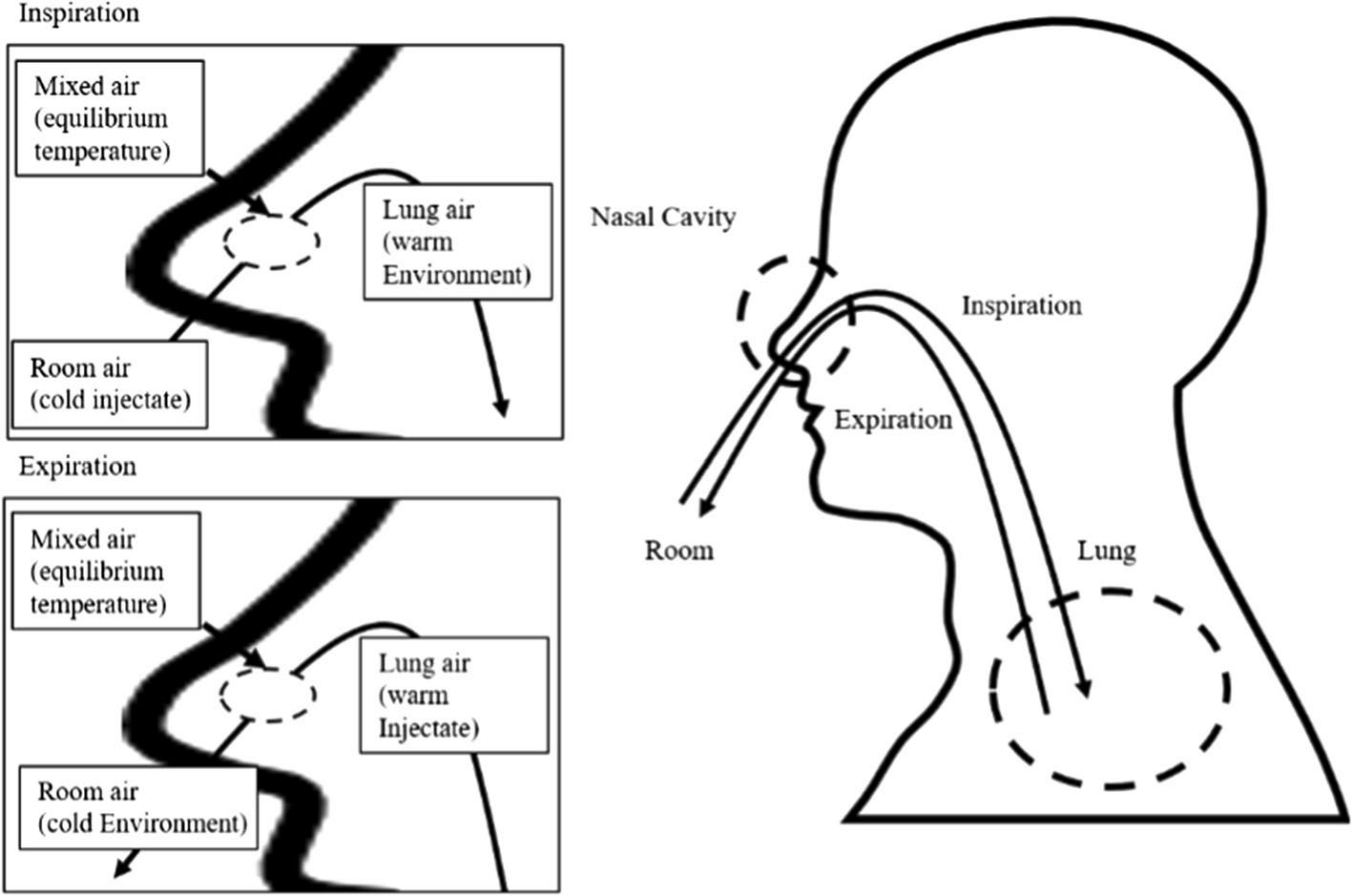
Model concept: this picture illustrates the approach of the proposed model. For the case of inspiration, cold room air acts as the injectate and warm lung air acts as the environment. For the case of expiration, warm lung air acts as the injectate and cold room air acts as the environment. The thermal energy of the injectate and environment are exchanged in the nasal cavity

In this paper, the respiratory system is simplified to a single closed system. In the proposed closed system, cold room air from outside flows through the nasal cavity and passes through the airways into the lungs (inspiration). This means that the flow of air is warmed in the lungs and passes back through the airways and then exits through the nasal cavity (expiration). In the closed system, the temperature does not change with the flow of the air area outside the nasal cavity and inside the lung. Thus, the temperature change due to airflow occurs only in the nasal cavity. Figure 5 shows a respiration model including the concepts of inspiration and expiration. The model shown in Fig. 5 can describe the relationship between respiration and thermal energy in an ideal situation.

### Formula

**Fig. 6:**
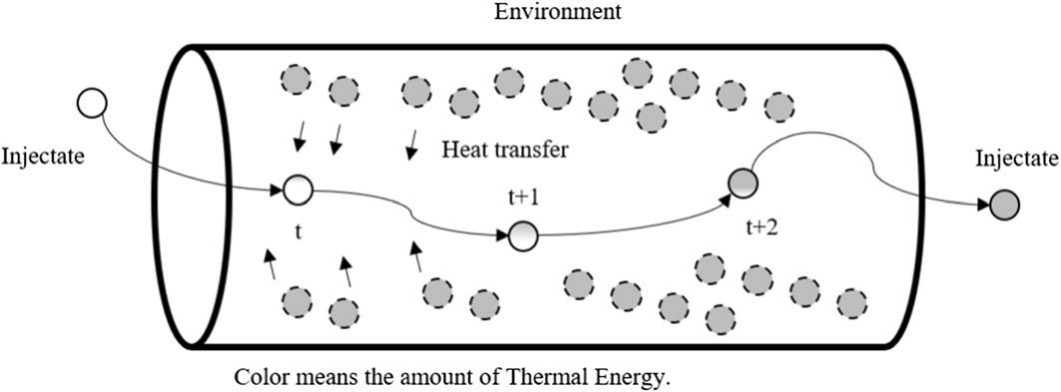
This image shows how the injectate obtains thermal energy from the environment. The line describes the time sequence of the injectate. The gray levels indicate the amount of obtained thermal energy

In principle, the relationship between respiration volume and thermal energy in the proposed closed system is identical to that of the thermodilution of cardiac output and blood thermal energy. Table 2 shows the comparison of the variables used in cardiac output and respiration models. Air warmed in the lungs is mixed with external nose air in the nasal cavity. This produces a mixture that is similar to that of the injectate and blood in thermodilution, and the temperature change of the mixture can be measured at a suitable point in the nasal cavity. Information about measured temperature–time values and amounts of temperature change in heat content due to air molecule interactions can be used to calculate the volume rate of airflow in a manner similar to thermodilution. In the case of expiration, the injectate is the warm air heated in the lung, and the environment is the external nose air. Inspiration is the opposite. The environment that exchanges temperature with the injectate in the closed system maintains homeostasis and always maintains a constant temperature. In other words, the environment provides or receives infinite energy for the injectate. Injectates interacting with the environment create a mixture by mixing with the environment. Depending on the state of the injectate, this mixture alters the heat energy it contains, and by observing the heat energy of the mixture, it is possible to calculate the thermodiluted respiration volume. Figure 6 is a conceptual image of how the injectate and environment exchange thermal energy in the nasal cavity. The thermal energy properties of the mixture caused by the injectate were monitored.

**Table 2:** Thermodilution model comparative overview difference

Cardiac output	Respiration volume
Injectate	Warm lung air (expiration) Cold nose air (inspiration)
Blood	Warm lung air (inspiration) Cold nose air (expiration)

The injectate obtains energy from the environment during inspiration and loses energy from the environment during expiration. The following expression describes the expiration condition, while the formula for the inspiration condition is described in Appendix A. Closed system is no change in the total energy of the thermal energy. It is assumed that the amount of heat lost in one mass is the same as the heat gained in another mass, as shown in Eq. (1):

1 $${m}_{N}{s}_{N}\left({T}_{N}-{T}_{E}\right)={m}_{L}{s}_{L}\left({T}_{E}-{T}_{L}\right),$$

where ${m}_{L}$[kg] and ${m}_{N}$[kg] are masses, ${s}_{L}$ [J/kg∙K] and ${s}_{N}$[J/kg∙K] are the specific heat of masses, and ${T}_{L}$ [K] and ${T}_{N}$ [K] are the initial Kelvin temperatures. ${T}_{E}$ is the equilibrium Kelvin temperature of the mixture air. In respiratory condition, ${T}_{L}$ is the lung temperature at the end cycle of inspiration, ${T}_{N}$ is the nostril temperature at the end cycle of expiration. ${T}_{E}$ is the equilibrium temperature at lung–nostril track. For continuous injection, Eq. (1) can be changed to Eq. (2):

2 $${M}_{N} {s}_{N}\left({T}_{N}-{T}_{E}\right)={M}_{L}{s}_{L}\left({T}_{E}-{T}_{L}\right),$$

where *M* [kg/s] is the mass flow rate, and N and L represent nose and lung air, respectively. For the case of a single injection, Eq. (2) can be changed to Eq. (3):

3 $${M_{N}} \cdot {t} \cdot {s_{N}} \left( {{T_{N}} - {T_{E}}} \right) = {m_{L}}{s_{L}} \left( {{T_{E}} - {T_{L}}} \right)$$

The heat-balance equation for the case of a single injection, Eq. (3), is where $t$ [s] is the “transit time” of the air past the detection point.

The value of $t$ is, in the limit, infinite and obviously arbitrary. It is converted to a tidal volume estimate using the thermal energy analog of typical thermodilution.

It should be realized, however, that the “warm” injectate, and the “cold”, shared by lung and nose air is ${m}_{L}{s}_{L}\left({T}_{N}-{T}_{L}\right)$.

Tidal volume estimations using thermal energy cannot measure the indicator quantity without reference to nostril temperature. Therefore, $\left({T}_{N}-{T}_{L}\right)$ can be considered constant.

$\Delta {T}_{R}\left(t\right)$ is the temperature–time function by using Meier and Zierler’s [14] general treatment, and $\Delta {T}_{R}\left(t\right)$ is proportional to the transit times’ frequency distribution for the labeled air, $\Delta {T}_{R}\left(t\right)$ is ${T}_{N}-{T}_{E}.$

4 $${m}_{L}{s}_{L}\left({T}_{N}-{T}_{L}\right)= {\int }_{0}^{\infty }\Delta {T}_{R}\left(t\right){M}_{N}{s}_{N} dt+E.$$

$E$ is the integral constant. For the equation in $\Delta {T}_{R}\left(t\right)$, the temperature term of the left side of Eq. (4) is converted to a constant value based on the initial temperatures of the lung air and nose air. In addition, in volumetric terms,

5 $${V}_{L}{\rho }_{L}{s}_{L}\left({T}_{N}-{T}_{L}\right)= {\int }_{0}^{\infty }\Delta {T}_{R}\left(t\right){Q}_{N}{\rho }_{N}{s}_{N} dt+E,$$

where ${Q}_{N}$ [L/s] is the volume rate of nose airflow, ${V}_{L}$ [L] is the injectate (lung) volume, $\rho$ is the density. If the airflow exhibits a constant steady-state, ${Q}_{N}$ can be set as a constant, although some error is introduced by density variations. Physical constants, such as $\rho$ and $s$, vary depending on temperature and time, but these differences are very small for the same medium. In other words, in the special case that ${\rho }_{L}$ = ${\rho }_{N}$ and ${s}_{L}$ = ${s}_{N}$, is it not only unnecessary to know the values of $\rho$ and$s$, but they are also assumed to be constant. Therefore, $C=\left|\frac{{Q}_{N}}{{T}_{N}-{T}_{L}}\right|$, $D=\left|\frac{E}{{T}_{N}-{T}_{L}}\right|$, is constant. This can be written more simply:

6 $${V}_{L}= C{\int }_{0}^{\infty }\Delta {T}_{R}\left(t\right) dt+D.$$

Tidal volume is the amount of air that is inhaled or exhaled through the lungs with each respiration. Therefore, tidal volume can be calculated using either inspiration or expiration.

### *Measurements of *$\Delta {T}_{R}(t)$* using a thermal camera*

The nasal cavity, which is the inner part of the nose, is the location where air passes during respiration. When inhaling, cold air from outside the body enters. When exhaling, warm air from the lungs is released. In other words, there is a temperature gradient in the inner space of the nose. Heat transfer in the air of the nasal cavity changes the temperature of the structure that is observed by the thermal camera. The parameter $\Delta {T}_{R}\left(t\right)$ should be measured in the nasal cavity of the respiratory system. However, the nasal cavity area is difficult to measure. In an ideal closed system, the total amount of thermal energy in the nasal cavity remains constant, and only the inflow and outflow of air exchange thermal energy, so there is no outflow of thermal energy outside the body. In reality, however, this change in temperature proportionally affects the change in temperature of the body tissues that constitute the nasal cavity, which can be measured by an external thermal camera. In this paper, $\Delta {T}_{R}\left(t\right)$ is inferred by measuring the temperature of the affected skin based on the theory that the temperature of the nasal cavity affects skin temperature [15]:

7 $$\Delta {T}_{R}\left(t\right)=\underset{S}{\overset{ }{\int }}\Delta {T}_{S}\left(t\right) ds.$$

**Fig. 7:**
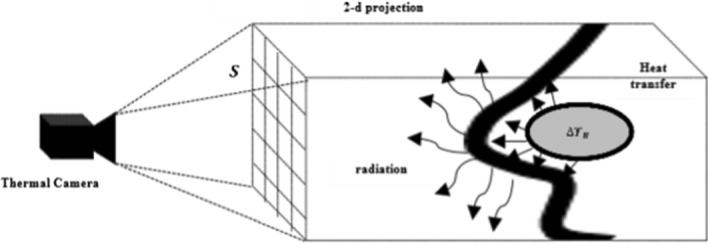
Measurement concept: this picture illustrates the measurement of thermal change. This figure show how temperature changes within nasal cavity are measured with thermal camera

A thermal imaging camera can indirectly measure the skin temperature and can be used to provide temperature data for a two-dimensional plane. $\Delta {T}_{S}\left(t\right)$ is skin temperature change affected by the nasal cavity. In this case, the area captured by the thermal imaging camera is projected onto a 2-D plane, so it is not possible to express all temperatures of the actual skin surface, but it is assumed that the differences are insignificant. Therefore, the surface region can be expressed in 2-D plane with $\mathrm{S}\to {\mathbb{X}}\left(x,y\right) \in {R}^{2}.$ In Eq. (5), the term ${Q}_{N}$ can be changed to the value corresponding to each 2-D pixel coordination as unit volume rate of respiration flow ${q}_{N}$. Figure 7 is a conceptual image of how temperature changes within the nasal cavity are measured with the thermal camera. Therefore, measurements of ${V}_{L}$ using a thermal camera can be expressed as Eq. (8):

8 $${V}_{L}= C{\int }_{0}^{{\mathrm{t}}_{tidal} } {\iint }_{S}\Delta {T}_{R}\left(t,{\mathbb{X}}\right) dtd{\mathbb{X}}+D.$$

### Estimation of thermodiluted relative tidal volume in a practical system

In general, tidal volume refers to the volume of air that has moved in inspiration or expiration during stable normal respiration. It is calculated as the amplitude of the peak-to-peak volume displacement [16].

In this paper, to calculate tidal volumes while simultaneously considering both inspiration and expiration, tidal volumes are calculated using the mean inspiration tidal volumes and expiration tidal volumes. Therefore, the equation for calculating the *N*th tidal volume (TV) is as follows:

9 $${TV}_{N}=\frac{{V}_{{L}_{N}}\left({t}_{2}\right)-{V}_{{L}_{N}}\left({t}_{1}\right) }{2}+\frac{{V}_{{L}_{N}}\left({t}_{2}\right)- {V}_{{L}_{N}}\left({t}_{3}\right) }{2},$$

where ${t}_{1}$ refers to the beginning of respiration(start of inspiration), ${t}_{2}$ refers to the end of inspiration(start of expiration), and ${t}_{3}$ refers to the end of respiration(end time of expiration).

In an ideal closed system, tidal volume can be calculated with Eq. (5). However, there are various artifacts in practical systems.

The tidal volume contains variable artifacts in practical systems. The equation for the thermodiluted tidal volume of a practical system when individual calibrating the two-factor types is as follows:

10 $${\mathrm{TV}}_{\mathrm{RESP}\_{\mathrm{Practical}}_{N}}=\mathrm{\alpha }\cdot {T{\mathrm{V}}_{\mathrm{N}}}+\upbeta$$

$\mathrm{\alpha }$ and $\upbeta$ are caused by the differences in heat-transfer coefficients on the skin surface each time. The term ${k}_{S}$ has a different coefficient for each skin and time, which cause differences. In addition, when the surface of the skin, which is a 3-D curved surface, is imaged with a thermal camera, the result is projected onto a 2-D plane and thereby includes the effect of the distorted heat-transfer coefficient. Also, error includes the shape, size, movement, and angle of the nostril to be measured by the camera. The proposed formula can estimate the relationship between the temperature change caused by respiration and the relative tidal volume of respiration in way of semi-analytical approach.

### Data acquisition

**Fig. 8:**
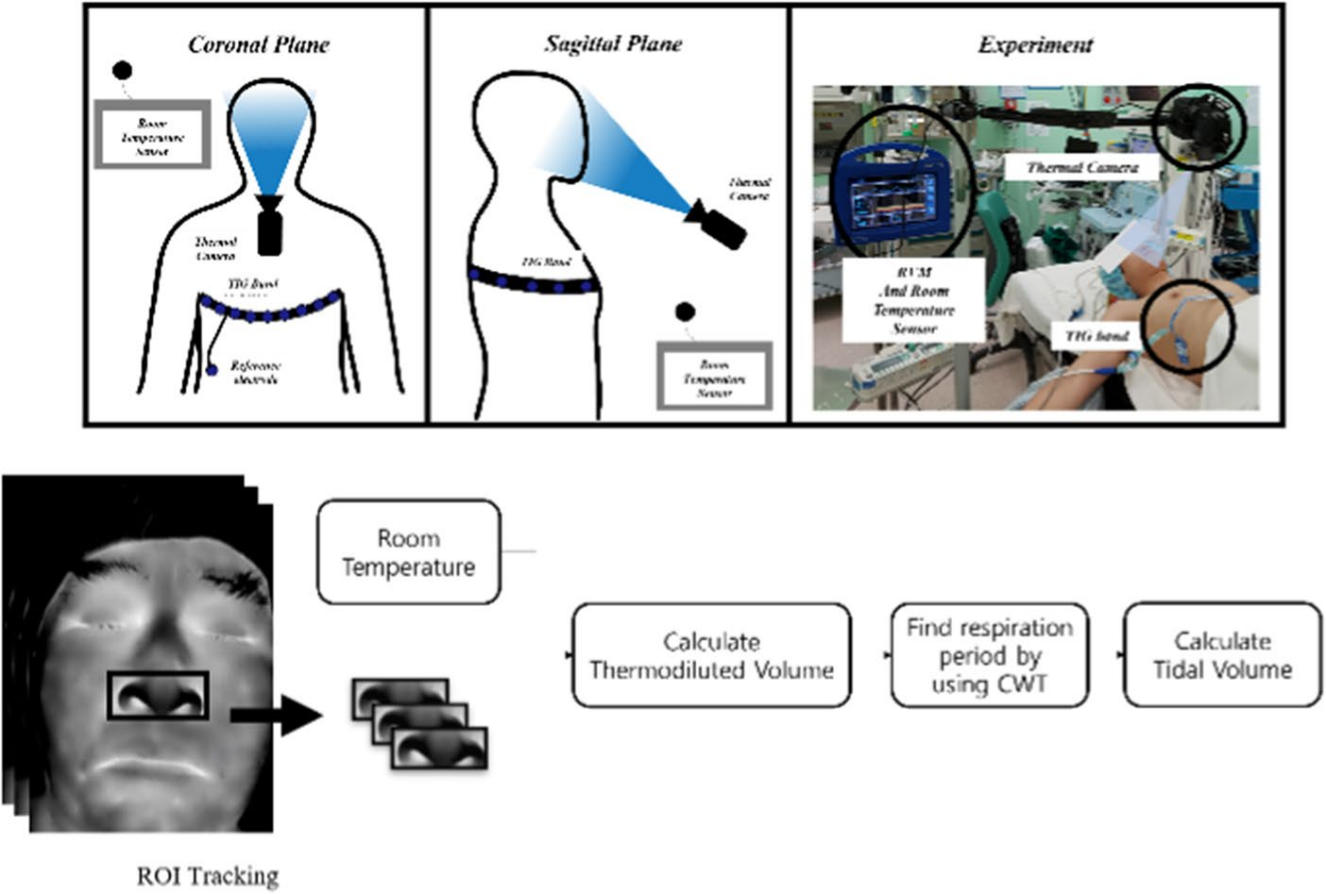
Method flowchart

In this paper, this study compared the linearity of the mean tidal volume that is estimated by the proposed formula and that obtained from the TIG as the golden standard signal. Then, this study compared the fit of the model to experimentally validate the derived formula. Figure 8 shows the method flowchart.

The study was performed at Severance Hospital from February 2017 to July 2017. This study was conducted in accordance with principles of Good Clinical Practice and was approved by the Institutional Research Board of Severance Hospital (Reference Number. 1–2016-0008); all patients gave informed consent. The study was registered at ClinicalTrails.Gov with the number NCT02993497.

To monitor real-time respiration, the TIG data acquisition (used as the reference) is also used as a noninvasive RVM machine [17, 18]. The RVM noninvasively measures the respiration volume based on changes in the electrical impedance of the chest. The impedance signal is measured by using electrodes attached to the sternal notch, xiphoid process, and mid-axillary sites. Changes in impedance are calculated based on the changes in impedance that are related to respiration volumes for the first 30 s. Based on this, the relative tidal volumes (percentage value) are calculated and recorded every 5 s using an internal algorithm [19]. In this paper, linear interpolation of TIGs was performed to use the same sampling rate as the thermal camera.

To determine respiration volumes with a thermal imaging camera, the absolute temperature distribution across the subject’s nostrils was simultaneously acquired. The experiment was performed with healthy (no respiratory symptoms, age: 30 ~ 39) volunteers eight subjects. The subjects have experimented with little movement while respiration. The operating room temperature ($${T}_{R})$$ in the experimental environment was sampled at 1 kHz using BIOPAC MP 150TM. Operating room temperature ($${T}_{R})$$ was between 20.5 and 23.5 degrees Celsius. All participants were maintained in a stable for 2 min after receiving consent. The camera (FLIR T-420, FLIR Systems, Inc.) acquired images of a subject from 50 cm above the face who was lying face up at the same time as surgery. The FLIR T-420 is a thermal camera that uses LWIR (7.5 to 13 μm). The spatial resolution of the thermal imaging camera was 320 × 240, and the noise equivalent temperature difference (NETD) was 0.05 Kelvin. The thermal image included the nasal cavity and nostrils and was acquired at 10 frames per second. It was acquired using a program developed with FLIR's SDK (C +  + Active X) in an I7-5500U, 8 GB RAM environment, and the absolute Kelvin temperature was stored as a CSV file with data to two decimal places. In the case of ROI registration, KLT Tracking was used [20]. The nostril region was found using J Fei’s method [9]. In addition, SpO2 was simultaneously measured. When hypoxemia (SpO2 < 90%) was detected, the subject was immediately awakened to induce spontaneous breathing and additional oxygen was provided through a facial tent mask.

### Find respiration period by using continuous wavelet transformation (CWT)

**Fig. 9:**
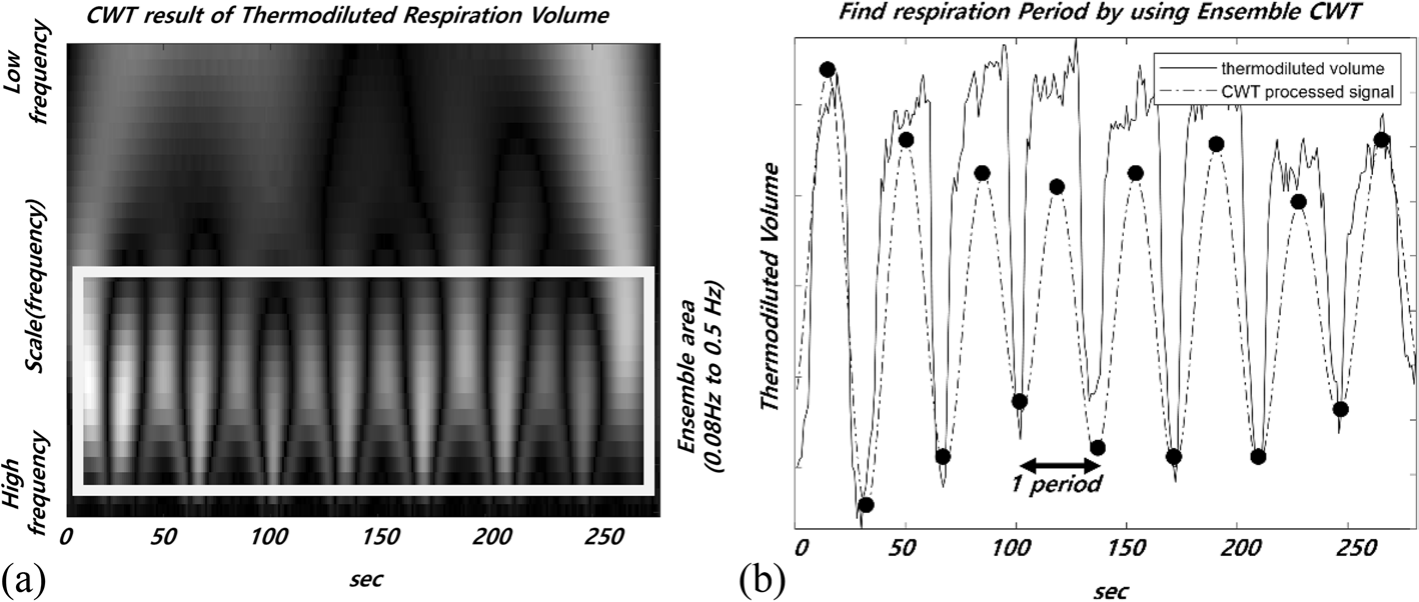
**a** CWT result of thermodiluted volume signal with the ‘mexh’ mother wavelet. **b** Shows the thermodiluted volume signal and respiratory index that were extracted using the ensemble CWT. Each index is used in Eq. (12) for calculating the thermodiluted tidal volume

A continuous wavelet transform has good strength for identifying the characteristics of a changing nonstationary signal. A typical nonstationary signal is a biosignal, and the breathing signal also has the characteristics of a biosignal. Thermodiluted volumes were extracted using Eq. (10) for the temperature data obtained through the alignment and operating room temperature. Because the extracted signal is not sufficiently stable to determine the breathing cycle, it is difficult to clearly determine the breathing period. To identify a clear breathing period, the volume signal was transformed through a CWT. To consider all breathing signals in various frequency bands in the signal when converting a signal with a CWT, the frequency band for breathing that was included in the CWT ensemble was extracted. The frequency band used was 0.08 Hz to 0.5 Hz, which includes both bradypnea and tachypnea and covers all of the respiratory bands for which humans can breathe. The converted signal represents a stable breathing period index. The mother wavelet used included “mexh”, which is a function of quadratic Gaussian differential form and is similar to the general form of the respiratory signal. Figure 9a shows CWT overview result. Boxed parts mean 0.08 Hz to 0.5 Hz frequency bands. Figure 9 b shows the calculated thermodiluted volume signal and respiratory index that were extracted using the ensemble CWT. The thermodiluted volume period for calculating tidal volume by applying Eq. (10) according to each breathing cycle was extracted from the thermodiluted volume signal by referring to the respiratory index that was extracted by the continuous wavelet transformation(CWT). According to the TIG analysis method that uses the calculated tidal volume as a reference value, the similarity was measured by calculating a 30 s moving window average. All data had margins at both ends for the 30 s average to minimize data interference. Each data was compared through a normalization process.

## Data Availability

Not applicable.

## Appendix

The injectate of inspiration is cold room air, and the environment is lung air that is heated inside the body. There is no change in total calories of the heat content in the closed system. It is assumed that the heat lost in one mass is the same as the heat gained in another mass, as shown in Eq. (11):

11 $${m}_{L}{s}_{L}\left({T}_{L}-{T}_{E}\right)={m}_{N}{s}_{N}\left({T}_{E}-{T}_{N}\right),$$

where ${m}_{L}$[kg] and ${m}_{N}$[kg] are masses, ${s}_{L}$ [J/kg K] and ${s}_{N}$ [J/kg K] are the specific heat of masses, and ${T}_{L}$ [K] and ${T}_{N}$ [K] are the initial kelvin temperatures. ${T}_{E}$ is the equilibrium kelvin temperature of the mixture air. For continuous injection, Eq. (11) can be changed to Eq. (12):

12 $${M}_{L}{s}_{L}\left({T}_{L}-{T}_{E}\right)={M}_{N}{s}_{N}\left({T}_{E}-{T}_{N}\right),$$

where *M *[kg/s] is the mass flow rate, and N and L represent nose and lung air, respectively. For a single injection, Eq. (12) can be changed to Eq. (13):

13 $${M}_{L} \cdot t \cdot {s}_{N}\left({T}_{L}-{T}_{E}\right)={m}_{N}{s}_{N}\left({T}_{E}-{T}_{N}\right).$$

It should be realized, however, that the “cold” injectate, that is, the “warm”, which is shared by the nose and lung air, is ${m}_{N}{s}_{N}\left({T}_{L}-{T}_{N}\right)$.

$\Delta {T}_{R}\left(t\right)$ is the temperature–time function. Equation (13) can be changed to Eq. (4). $\Delta {T}_{R}\left(t\right)$ is ${T}_{L}-{T}_{E}.$

14 $${m}_{N}{s}_{N}\left({T}_{L}-{T}_{R}\right)= {\int }_{0}^{\infty }\Delta {T}_{R}\left({t}_{INS}\right){M}_{L}{S}_{L} dt+E.$$

$E$ is the integral constant. For the equation in $\Delta {T}_{R}\left(t\right)$, the temperature term of the left side of Eq. (14) is converted to a constant value that is based on the initial temperatures of the lung air and room air. In addition, in volumetric terms,

15 $${V}_{N}{\rho }_{N}{s}_{N}\left({T}_{L}-{T}_{N}\right)= {\int }_{0}^{\infty }\Delta {T}_{R}\left(t\right){Q}_{L}{\rho }_{L}{s}_{L} d\mathrm{t}+\mathrm{E},$$

where ${Q}_{L}$ [L/s] is the volume rate of lung airflow, ${V}_{N}$ [L] is the injectate (Nose) volume, $\rho$ is the density. If the airflow is at a constant steady-state, ${Q}_{L}$ can be set as a constant although some error is introduced by density variations. Physical constants such as $\rho$ and $s$ vary depending on temperature and time, but the differences are very small for the same medium. In other words, for the special case that ${\rho }_{L}$ = ${\rho }_{N}$ and ${s}_{L}$ = ${s}_{N}$, not only is it unnecessary to know the values of $\rho$ and $s$, but they are also assumed to be constant. Therefore, $C=\left|\frac{{Q}_{N}}{{T}_{N}-{T}_{L}}\right|$, $D=\left|\frac{E}{{T}_{N}-{T}_{L}}\right|$, is constant. Thus, Eq. 15 can be more simplified to:

16 $${V}_{N}=\mathrm{ C}{\int }_{0}^{\infty }\Delta {T}_{R}\left(\mathrm{t}\right) d\mathrm{t}+\mathrm{D} \left(\mathrm{inspiration}\right).$$
